# Sequence-Based Characterization of Intratumoral Bacteria—A Guide to Best Practice

**DOI:** 10.3389/fonc.2020.00179

**Published:** 2020-02-21

**Authors:** Sidney P. Walker, Mark Tangney, Marcus J. Claesson

**Affiliations:** ^1^Cancer Research at UCC, University College Cork, Cork, Ireland; ^2^SynBioCentre, University College Cork, Cork, Ireland; ^3^APC Microbiome Ireland, University College Cork, Cork, Ireland; ^4^School of Microbiology, University College Cork, Cork, Ireland

**Keywords:** cancer, low biomass, FFPE, sequence analysis, microbiome

## Abstract

Tumors are hospitable environments to bacteria and several recent studies on cancer patient samples have introduced the concept of an endogenous tumor microbiome. For a variety of reasons, this putative tumor microbiome is particularly challenging to investigate, and a failure to account for the various potential pitfalls will result in erroneous results and claims. Before this potentially extremely medically-significant habitat can be accurately characterized, a clear understanding of all potential confounding factors is required, and a best-practice approach should be developed and adopted. This review summarizes all of the potential issues confounding accurate bacterial DNA sequence analysis of the putative tumor microbiome, and offers solutions based on related research with the hope of assisting in the progression of research in this field.

## The Tumor Microbiome: Current Status and Future Challenges

The existence of a tumor bacterial microbiome is still a contentious concept, but an increasing number of articles are being published exploring this novel habitat, and simultaneously exploring the possible effects these bacteria could have. To inform the direction such research will take in the future, it is important to take stock of the research carried out to this point to learn from past mistakes, and similar analyses in relevant fields. Research to date has focused on two key questions; what is there, and what does it do? This has involved comparing the microbiota of malignant and non-malignant breast tissue (including non-cancer patient) in the original studies (1–3). Subsequent studies examined potential causative links between bacteria and their host tumors, or assessing their metabolic activity, for example their effect on chemotherapeutics (4, 5). These concepts have important potential in cancer care, in terms of treatment regime, diagnosis or prevention, but rely on the field developing a thorough understanding of the microbial-related tumor microenvironment.

The key hurdles in accurately characterizing these environments are outlined as follows.

- Tumor samples are regions of known low microbial biomass, a feature which complicates any metagenomic analysis. This review will include suggested methodologies for bioinformatic analysis of tumors, and also of low biomass samples in general. Linked to the issue of low biomass, tumor samples present an extremely high ratio of host to bacterial DNA, which can lead to bias in amplicon based sequencing strategies such as 16S rRNA sequencing, and can make whole genome sequencing impossible without a microbial enrichment strategy (6).
- A further problem relates to the quality and quantity of patient tumor-related samples. Sourcing high numbers of aseptically-collected samples to enable statistical power is challenging, due to potential impact on standard of care, the workload of healthcare professionals, and competing requirements of the hospital diagnostic and other research teams for a limited amount of sample. A resource with potential for higher sample throughput for tumor metagenomics analysis is formalin-fixed paraffin-embedded (FFPE) tissues, the international gold standard for tissue sample storage. A proof of concept study recently showed that FFPE tissues provided a reliable source of germline and malignant human DNA (7). It is hoped that FFPE tissues can provide reliable bacterial DNA also, once the proper precautions are taken, not least distinguishing contamination inherent to this biobanking process. As with the low biomass characteristic, FFPE tissues would also present challenges to any bioinformatics analysis.
- When performing library preparation and bacterial DNA sequence analysis to investigate the tumor microenvironment, the issues raised in (i) and (ii) manifest in a number of ways. Introduced environmental contamination is likely to be inherent given the sampling process, which, given the low biomass nature of this tissue, has the potential to obscure tumor-originating bacteria. Similarly, there are other issues associated with low biomass such as PCR bias caused by the high ratio of host to bacterial DNA. If FFPE samples are used, errors in the sequence data will occur due to DNA damage during the formalin fixation process (8, 9).

**Figure 1:**
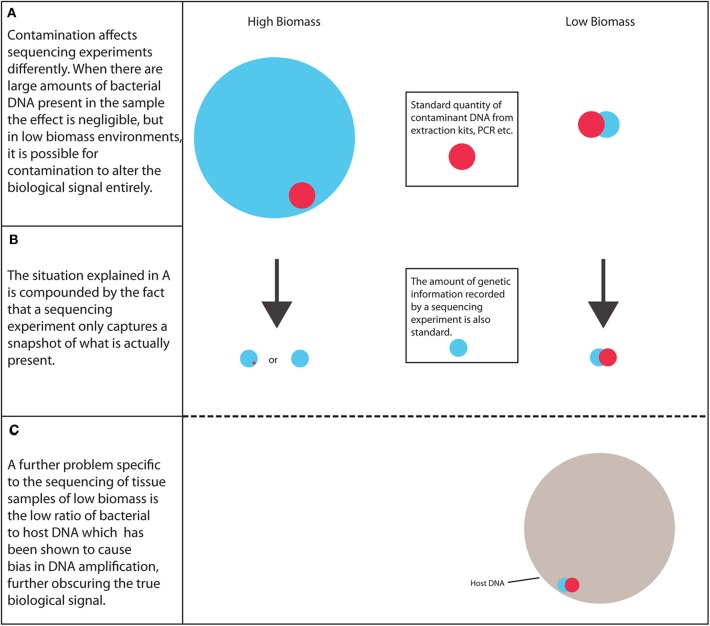
The scale of the problem. Low biomass environments are considerably more susceptible to biological signal alteration arising from contaminant DNA than high biomass samples, along with the increased likelihood of PCR bias.

In summary, as more research is carried out into the tumor microbiota, it is important to address the many potential pitfalls involved to ensure that these environments are reliably characterized, the scale of the problem is shown in Figure 1. The credibility of this field and other low biomass fields has been affected by recent publications highlighting methodological mistakes in previous research characterizing the microbiome of tumors and other low biomass environments (10). Therefore, a robust strategy needs to be established to ensure that future results are as reliable as possible.

## Research on the Tumor Microbiome to Date

The recent work characterizing the microbiomes of solid tumors is outlined in Table 1 below. Due to the challenges posed in characterizing the tumor microbiome, it is likely that some or all of the studies referenced have been negatively impacted in some way, reducing their accuracy. This caveat must be kept in mind when assessing the results, and reinforces the need for the introduction of a best practice methodology to make future research more reliable.

**Table 1:** Tumor sites with suspected bacterial communities.

**Tumor site**	**Description**	**Bacterial community**	**References**
Breast	Tumor tissue has microbial signature similar to surrounding tissue Tumor adjacent tissue significantly different to non-cancer patient breast tissue	*Enterobacteriacae* spp. (Proteobacteria), *Gammaproteobacteri*a spp. (Proteobacteria), *Acinetobacte*r spp. (Proteobacteria), *Bacillus* spp. (Firmicutes), *Staphylococcus* spp. (Firmicutes), and *Lactococcus* spp. (Firmicutes) Bacteria found in healthy breast tissue: *Micrococcus* spp. (Actinobacteria) and *Prevotella* spp. (Bacteroidetes), and to lesser extend *Lactococcus* spp. and *Gammaproteobacteria* also found in cancer-related tissue	(1–3)
Pancreatic Ductal Adenocarcinomas (PDAC)	*The* cancerous pancreas has a more abundant microbiota than healthy control The gut microbiome responsible for ~25% of the tumor microbiome, but was absent from normal adjacent tissue	Enterobacteriaceae, Pseudomonadaceae and to a lesser extent *Streptococcaceae, Staphylococcaceae* and *Micrococcaceae*	(5, 11)
Prostatic cancer	Tumor and adjacent tissue significantly different from non-tumor prostate specimens	Actinobacteria, Firmicutes and Proteobacteria, Lactobacillales and *Streptococcaceae* significantly elevated in healthy samples, *Staphylococcacea* in tumor and peritumor	(12)
Colorectal cancer	CRC tumor tissue has microbial signature similar to surrounding tissue CRC-associated microbiota profiles, both malignant and non-malignant, differ from healthy controls	*Bacteroides, Roseburia, Ruminococcus, Oscillobacter*, and oral pathogens such as *Fusobacterium* elevated in CRC patients	(13)
Others	Ovarian and lung cancer tumor microenvironments have also been characterized		(14, 15)

## Is There an Aetiological Relationship Between Tumors and Bacteria?

Considerable research has been conducted to demonstrate the link between the microbiota and a variety of proximal and distal cancers. Some associations were found to be directly causative, such as *H. pylori* and Gastric Adenocarcinoma (16). In other circumstances, reports suggesting certain bacteria being elevated in specific instances of cancer along with a variety of potential mechanisms for causing/progressing the aforementioned cancer make a strong case, even if the final confirmation has yet to be found. An example of this is the constantly developing picture of the role *Fusobacterium* plays in colorectal cancer (17). Mycoplasma infection has also been shown to transform normal lung cells, affecting cell proliferation and differentiation (18). Research has also been carried out suggesting that some microbiome constituents confer protection against tumor formation. For example, short chain fatty acids of microbial origin such as butyrate and propionate can inhibit tumor cell histone deacetylases, and pyridoxine, also of bacterial origin can help promote tumor surveillance by the host immune system (19). An interesting case is that of *H. pylori*, which as mentioned, has been proven to cause gastric adenocarcinoma, but may also have a protective role against eosophogeal adenocarcinoma (20).

In many tumors, it may be that bacteria are simply opportunistic inhabitants (21, 22). Circulating blood is generally considered to be sterile. However, due to the hospitable nature of tumors to bacterial growth, circulating bacteria could be colonize tumor tissue before replicating locally. Although bacteria may enter the blood stream transiently due to surface wounds, human trials have found that efficient tumor colonization only resulted from administration of high concentrations of bacteria intravenously. The conditions required for this to occur naturally would require a blood bacteria concentration high enough for tumor colonization, but not so high as to induce septic shock (23). A potentially more persistent source would be bacterial translocation from the intestine, which we and others have reported (23, 24). While a translocation mechanism for non-pathogenic bacteria in this context is as yet unexplained, there are several factors that may independently or cumulatively cause bacterial translocation to occur–intestinal microbial imbalance, increased permeability of the intestinal mucosa, and host immunodeficiency (25).

As mentioned previously, tumors are uniquely amenable to bacterial colonization, and unlike healthy tissues, conceivably provide a refuge for any circulating bacteria, including non-invasive species. A collection of phenotypes unique to tumors which have been proposed to explain the phenomenon of selective tumor colonization by bacteria are as follows;

- Angiogenesis associated with tumor growth is an imperfect process, resulting in disorganized or “leaky” vasculature. This could allow circulating bacteria to embed themselves in the tissue.
- Tumors are immune privileged regions of the body. This characteristic means that bacteria which may be cleared by the host immune system at other body sites are able to proliferate within tumors.
- Many solid tumor regions are hypoxic, this lower level of oxygen compared with healthy surrounding tissue provides an environment that suits the proliferation of facultative and anaerobic bacteria.
- Necrotic regions within the tumor are nutrient rich, promoting bacterial proliferation.

**Figure 2:**
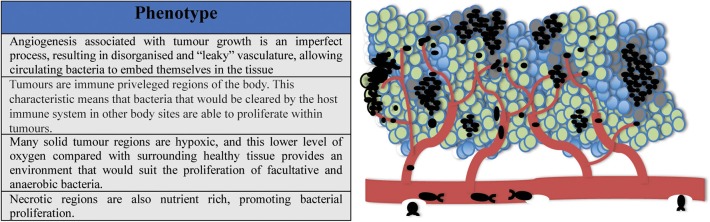
Tumors are uniquely hospitable environments for bacteria. (i) Leaky vasculature allows circulating bacteria to embed in tumor tissue; (ii) Tumors are immune privileged regions; (iii) Solid tumors possess low oxygen regions suitable for the proliferation of facultative and anaerobic bacteria; (iv) High-turnover regions of tumors can be nutrient rich, promoting bacterial growth.

These phenotypes are shown in Figure 2.

## What is the Significance of Endogenous Bacteria Residing Within Tumors?

Beyond ongoing research into any causal relationships between bacteria and tumors, there are several other benefits to fully understanding these habitats. Understanding what bacteria colonize tumors could help with the development of more personalized or targeted treatment regimens for many tumors, maximizing effect on the tumor and minimizing the impact on the patient. A number of potential influences (both positive and negative) of resident intratumoral bacteria on tumor growth and responses to treatments have already been proposed by us and others, and include effects on therapeutics, potential cross-talk between cancer cells and bacteria, and the potential for intratumoral bacteria to mediate therapy.

### Effect on Host Immune Response

Very little data exist on effects of bacteria residing within tumors on the host immune response. This has been researched most comprehensively in patient pancreatic cancer, but as of yet no consensus has been reached. A recent study concluded that endogenous bacteria facilitate oncogenesis by local suppression of the innate and adaptive immune response (11), while other research has identified distinct microbial profiles within tumors of patients with increased PDAC survival rates, indicating that bacteria or groups of bacteria may be associated with long term survival (26).

### Biotransformation of Drugs

Bacteria have a long established ability to transform organic chemicals via endogenous enzymes, so much so that an industry exists to harness this ability (27). We were the first to report that a variety of unmodified bacteria found in tumors, such as *E. coli*, with natural levels of endogenous enzymes can either positively or negatively affect the efficacy of various chemotherapeutics, such as gemcitabine, as evidenced by *in vitro* and *in vivo* cancer models (4). This knowledge, coupled with an accurate characterization of the tumor microbiome, may facilitate a more targeted approach to chemotherapy, for example, through modulation of a tumor's microbiota. Bacteria which are known to inhibit the action of therapeutics might be selectively removed with antibiotics, while bacteria which enhance these therapeutics could be introduced to the microenvironment prior to proceeding with the treatment to improve its efficacy.

### Bacteria as Therapeutic Agents

Given their unique capacity for selective growth in tumor tissue, therapeutics may be locally produced within the tumor by administered engineered bacteria (28–34). Bacteria can also be engineered to “sense” their environment, using synthetic biology approaches, further increasing their therapeutic or diagnostic power (35–37). This notwithstanding, by analyzing the microbiota of tumors, it is possible that bacteria may be found that selectively colonize tumors or have a significant gradient increasing from other sites in the body to the tumor. The discovery of such bacteria would reduce the need for genetic engineering of bacteria for specificity as they would naturally colonize the tumor following systemic administration. Given current regulations regarding the use of genetically modified micro-organisms this would be an extremely useful feature in any candidate bacterium. However, it must be added that considerable challenges stand in the way of an approach such as this becoming a reality. Most notably the extremely narrow therapeutic index for the intravenous administration of bacteria for therapeutic purposes which provides a major hurdle to their routine administration (23).

## Biological Considerations

Formalin-fixed paraffin-embedded tissue (FFPE) represents a resource that, if correctly harnessed, could exponentially increase the sample sizes and sites available for tumor microbiota studies. Crucially, these do not have to be obtained at the time of surgery, like fresh frozen tissue, although both fresh frozen and FFPE tissues involve difficulties.

### Patient Sample Logistics

The realities of patient sample acquisition must be taken into account by researchers in this field.

- Sampling-related contamination e.g., from the patient, the operating theater, or the pathology lab (tissue handling and processing) must be considered in the design of research workflows (see later).
- Broad-spectrum antibiotic administration can be routine in many hospitals immediately prior to tumor resection operations. While interfering with the clinical standard of care is difficult, antibiotic administration should be considered and reported in such studies.
- An under-considered parameter is that tissue is heterogenous within a tumor, and bacterial profiles are likely to differ (quantity and quality) intratumorally, with some tumor tissue providing different growth conditions to other regions. Hence, typical pathologist-preferred tumor regions required for diagnosis (e.g., “margins”) may not accurately represent the endogenous bacterial community residing in the tumor. Histological and *in vivo* imaging data from our lab has indicated that anaerobic species in particular localize to less viable regions within the tumor, distal to any vasculature, albeit in mouse tissue (38, 39). This is complicated further by the presence of host immune cells such as neutrophils which have been shown to form a barrier separating the bacteria rich necrotic regions from viable cells (40).

### Low Biomass

Tumors represent low bacterial biomass samples. This poses a variety of challenges to the data generation process. This is a situation where the bacterial DNA that is the target of the study, is outnumbered by orders of magnitude by host DNA. Due to the targeted PCR amplification of bacterial DNA in 16S rRNA gene sequence analysis, this heavy ratio of host to bacterial DNA is commonly considered unimportant. This is not the case, with many studies demonstrating a reduction in PCR amplification efficiency in circumstances of high human nucleic acid and low bacterial 16S rRNA gene fragment copies, ultimately leading to sampling bias (41). Therefore, an effective host DNA depletion strategy is an important component of a 16s rRNA gene sequencing library preparation.

Commercial kits for microbial enrichment by host DNA depletion were recently compared by Marotz et al. (9). These included MolYsis, QIAamp, and lyPMA kits. All were found to significantly improve the microbial yield. lyPMA was the most effective, having a mean of 8–10% of reads aligning to the human genome, and MolYsis the least, with an average of 60% of reads aligning to the human genome. It is inevitable that the microbial DNA would also be affected. For example, the MolYsis approach is suspected to degrade bacteria with weak cell walls, or cell walls that have been previously weakened by exposure to certain antibiotics, so a balance between host depletion, and bacterial degradation must be found.

### Contamination

A recurring issue with low biomass samples is contamination, which poses a significant challenge in sequence analysis and interpretation. Often, the true microbiota can be masked by confounding bacterial DNA found in library preparation and DNA extraction kits. This feature is then often exacerbated by subsequent intensive amplification via PCR. Typical sources of contamination include environmental (surgery- and pathology-related), contaminants during the library preparation, and as has been recently described, contamination from within the extraction kit itself (10). Since Salter et al. published on this, there has been a general increase in awareness that reagent, laboratory and human contamination can have a serious impact on microbiome analysis (42). As water and soil associated bacteria are well-documented contaminants associated with DNA extraction kits and PCR reagents, some contaminants are easily identified if they make it through the sample preparation, sequencing, and bioinformatics contamination removal process. Genera such as *Bradyrhizobium*, which function in nitrogen fixation, are unlikely to be legitimate constituents of any human microbiome. The problem becomes more complex when sequences from *Escherichia* spp. and *Bacillus* spp. are found. Both have been shown to be artifacts of the library preparation process, but both are also common human pathogens (42). In 16S rRNA gene sequence analysis, taxonomic resolution to the species level is not always available, and never available in the instance of *Escherichia* spp., which compounds the problem.

### Summary of Contaminants Affecting 16s rRNA Gene Sequence Analysis

Table 2 shows a summary of recent articles addressing and discussing the problem of contamination in sequence analysis. It contains genera mentioned across all recent studies which include analysis of extraction and PCR kits, and also the ultra-pure water that is used in many kits and as a negative control.

**Table 2:** Previously identified bacterial contaminants.

**Phylum**	**Genus**
Actinobacteria	*Actinomyces, Aeromicrobium, Agrococcus, Arthrobacter, Atopobium, Beutenbergia, Bifidobacterium, Blastococcus, Brevibacterium, Candidatus, Planktoluna, Cellulosimicrobium, Clavibacter, Collinsella, Corynebacterium, Curtobacterium, Dietzia, Eggerthella, Geodermatophilus, Gordonia, Janibacter, Kocuria, Microbacterium, Micrococcus, Microlunatus, Patulibacter, Pilimelia*, *Propionibacterium, Pseudoclavibacter, Rhodococcus, Rothia, Slackia, Tsukamurella*
Bacteroidetes	*Alistipes, Bacteroides, Bergeyella, Capnocytophaga, Chryseobacterium*, *Cloacibacterium, Cytophaga, Dyadobacter, Flavisolibacter, Flavobacterium, Gelidibacter, Hydrotalea, Niastella, Olivibacter, Parabacteroides, Pedobacter, Porphyromonas, Prevotella, Wautersiella, Xylanibacter*
Deinococcus-Thermus	*Deinococcus, Meiothermus*
Firmicutes	*Abiotrophia, Anaerococcus, Anaerotruncus, Bacillus, Blautia, Brevibacillus, Brochothrix, Catenibacterium, Christensenella, Clostridium, Dialister, Dorea, Enterococcus, Erysipelatoclostridium, Eubacterium, Facklamia, Faecalibacterium, Fastidiosipila, Flavonifractor, Gemella, Geobacillus, Granulicatella, Halocella, Intestinibacter, Johnsonella, Lachnoanaerobaculum, Lachnoclostridium, Lachnospira, Lactobacillus, Listeria, Megasphaera, Moryella, Oscillospira, Paenibacillus, Papillibacter, Parvimonas, Peptococcus, Peptoniphilus, Pseudobutyvibrio, Pseudoflavonifractor, Quinella, Roseburia, Ruminococcus, Ruminosclostridium, Selenomonas, Solobacterium, Staphylococcus, Streptococcus, Trichococcus, Tumebacillus, Turicibacter, Tyzzerella, Veillonella*
Fusobacteria	*Fusobacterium, Leptotrichiaceae*
Proteobacteria	*Achromobacter, Acidovorax, Acinetobacter, Afipia, Alcanivorax, Alicycliphilus, Aquabacterium, Aquabacterium, Asticcacaulis, Aurantimonas, Azoarcus, Azospira, Beijernickia, Bosea, Bradyrhizobium, Brevundimonas, Burkholderia, Cardiobacterium, Caulobacter, Comamonas, Coprococcus, Craurococcus, Cupriavidus, Curvibacter, Delftia, Devosia, Diaphorobacter, Duganella, Enhydrobacter, Enterobacter, Eschericia, Geodermatophilus, Haemophilus, Herbaspirillum, Hoeflea, Janthinobacterium, Kingella, Klebsiella, Leptothrix, Limnobacter, Massilia, Matsuebacter, Mesorhizobium, Methylobacterium, Methylophilus, Methyloversatilis, Neisseria, Nevskia, Novosphingobium, Ochrobactrum, Oxalobacter, Paracoccus, Parasutterella, Pelomonas, Phyllobacterium, Polaromonas, Pseudomonas, Pseudorhodoferax, Pseudoxanthomonas, Psychrobacter, Ralstonia, Rhizobium, Rhodanobacter, Roseateles, Roseomonas, Rubellimicrobium, Ruegeria, Schlegelella, Serratia, Sphingobacterium, Sphingobium, Sphingomonas, Sphingopyxis, Stenotrophomonas, Sulfuritalea, Terrimonas, Thiohalocapsa, Undibacterium, Variovorax, Xanthomonas*
Tenericutes	*Mycoplasma*

## FFPE Tissue as a Source of Sample Tissue

With more developed screening methods and constantly improving medical care, particularly in the developed world, the size of tumors at the time of excision is rapidly reducing. The average size of a breast tumor has shrunk to <1 cm in diameter in the United States. As mentioned previously, this means fewer fresh “surplus to diagnostic” samples are available to research (43). Formalin fixation followed by paraffin embedding is the gold standard for preserving tissue samples after histological examination. FFPE blocks are stable at room temperature, and preserve the morphology and cellular details of tissue samples, along with the DNA. A unique problem when handling FFPE tissues is the degradation and mutation to which the DNA is subjected during the fixing and embedding process. FFPE blocks are undoubtedly a valuable resource due to the sheer quantity of samples available. However, there are several challenges involved in their effective use. Formalin fixation has been shown to cause cross-linking of histone-like proteins to DNA, DNA to formaldehyde adducts, and inter-strand DNA crosslinks (44). Generally, sequencing errors are caused by PCR mistakes, or miscalls during sequencing, but in a small set of circumstances, sequencing errors are caused predominantly by mutagenic DNA damage. These include ancient DNA from archaeological sites, circulating tumor DNA, and FFPE samples (45). The value of FFPE tissues as a sample type has begun to supersede the difficulty in their processing and analysis from a bacterial sequencing perspective. A recent study by Stewart et al. successfully used formalin fixed, paraffin embedded tissue to characterize the intestinal microbiota of pre-term infants with necrotising enterocolitis, despite some of their samples being almost 10 years old (46).

Although the strategies for minimizing and/or retroactively repairing this DNA damage mainly falls under the remit of the laboratory personnel carrying out the extraction and subsequent library preparation, there are some bioinformatics strategies that can be applied to lessen the impact of damaged DNA on the sequence data. Chen et al. proposed a method of scoring the extent of the errors in sequencing caused by DNA damage, called the Global Imbalance Value (GIV). This method is based on the directional adapters used in Illumina sequencing. The principle behind this is that because the majority of DNA damage only affects one base in a pair, DNA damage caused by oxidation, for example, could cause G-T transversion errors when the forward read of sequence data is mapped to a reference genome, but the reverse read would show the reverse complement of this, so C-A errors. This causes a “global imbalance” (45). A slight modification of this method would allow for the user to screen the reads generated by 16S sequencing of bacterial DNA within the tumor and in a process similar to the quality filtering already employed, only retain reads that had a GIV score below a certain threshold.

### Bacterial DNA Extraction From FFPE Samples

Despite these problems with using FFPE tissues for metagenomic analysis, there is a considerable history of bacterial identification in FFPE tissue in clinical settings, if not research settings (47). The QIAamp DNA FFPE Tissue kit is a purpose-built kit for the extraction of total genomic DNA from FFPE blocks produced by Qiagen. This kit compensates somewhat for damage caused by formalin fixation by including an incubation at elevated temperature following a proteinase K digestion. However, the kit does not take into account the oxidative damage that can be caused, or the extreme ratio of host to bacterial DNA, both of which can affect marker gene sequence analysis such as 16S rRNA gene sequencing (48).

**Figure 3:**
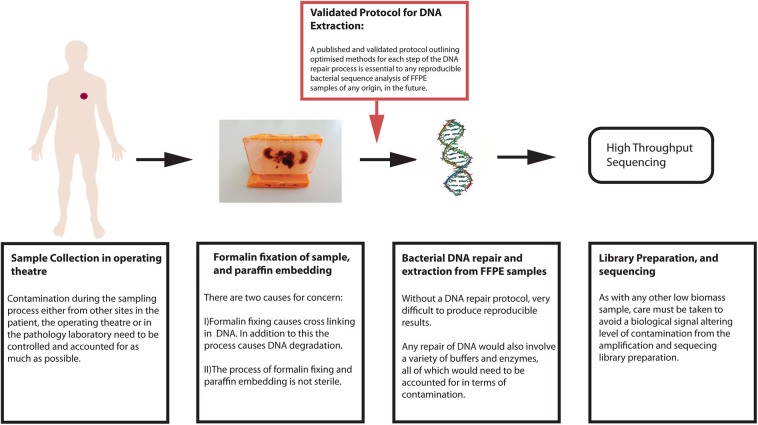
Workflow of biological considerations prior to bioinformatic sequence analysis.

If reliable characterization of the bacterial communities within tumors is to extend to FFPE samples, then a protocol for bacterial DNA extraction, repair and purification from these tissues is required to improve downstream analysis. A workflow of biological considerations for a sequencing experiment is shown here in Figure 3.

## Bioinformatic Aspects

### Detection of Microbial Communities

The two sequencing strategies employed in metagenomics analysis are whole genome sequencing and amplicon sequencing. Whole genome sequencing provides a high-resolution overview of all (or the most abundant, dependent on sequencing depth) DNA present in a sample. Bacterial genomes present will be characterized base by base, providing insights into bacterial taxonomy, function and rates of mutation, among other aspects. Host DNA present in the sample is also sequenced. Amplicon sequencing is a targeted approach allowing the targeting of specific regions within genomes, generally amplified by PCR. It is a two-stage process where primers are used to capture the target region, which is followed by high-throughput sequencing. Amplicon sequencing in bacterial microbiota studies typically targets the 16S rRNA gene subunit. This is the component of the 30 S small subunit adjacent to the Shine-Delgarno sequence, a region noted for its slow rate of evolution, containing nine “hypervariable regions” which can be used to differentiate between bacteria with varying degrees of effectiveness (49).

Whole genome sequencing has several advantages over 16S rRNA gene sequencing, such as increased species and strain level resolution, enhanced ability to detect rare species, and the ability to detect organisms in other kingdoms of life, such as viruses and fungi (50). At present 16S rRNA gene sequencing may be more technically suitable to metagenomics analysis of low biomass environments, in addition to being significantly more cost-effective. In a typical sequencing run of a non-tract biopsy in humans, 97% of the reads generated can be expected to align to a human reference genome (51). This makes it extremely expensive to get sufficient sequencing depth of the bacterial DNA present in a sample (51). As mentioned earlier, 16S rRNA gene sequencing is still affected by the low ratio of bacterial DNA, but to a lesser extent than whole genome sequencing methods. This can be improved upon by incorporating the previously mentioned host depletion strategies.

### Removal of Chimeric Reads

Chimeras arise as aborted extension products from earlier PCR cycles and can end up being taken up as a primer in a subsequent cycle. Undetected chimeric DNA sequences can be misinterpreted as novel species, particularly in 16S rRNA gene sequence analysis. Therefore, the number of PCR cycles can influence chimera formation (52). Given the low bacterial concentrations expected in tumor samples, the generation of chimeric reads is logically a significant cause for concern, and a robust protocol should be employed for their removal. Chimeras can be computationally identified and removed using one of a variety of programmes that fall into two groups. *De novo* methods usually work by identifying sequences which contain half of one abundant read and half of another, as evidenced by a difference in abundance between the start and the end of a sequence. Alternatively, reference-based methods compare reads identified to a curated database known to be chimera free, and attempts to find sequences that may have arisen from multiple samples (53). In this situation, where there is an elevated proportion of chimeras present, combining both methods would give the best chance of effective clearance of chimeras. Some of the most cited examples of chimera removal programmes across both categories include Chimera Slayer which is a referenced based method, Is.Bimera.Denovo which is the *de novo* chimera removal programme within the DADA2 pipeline, and UCHIME within the QIIME environment which has both reference based and *de novo* capabilities (53).

### Removal of Contamination

Two bioinformatics utilities have been developed recently, to retroactively solve this problem. SourceTracker, and Decontam (54, 55). These methods have different functionality but can be used in conjunction to remove contaminant taxa. The SourceTracker algorithm utilizes a Bayesian approach to provide an estimate of the proportion of contaminants that arise from possible source environments. Decontam looks for unusual relationships between DNA concentration in the original sample, and proportional abundance of sequence variants, and can add another layer by comparing samples with negative controls.

### Analyzing the Outputs

The traditional method of analyzing 16S rRNA gene sequencing data by clustering reads together based on a pre-defined threshold of similarity is no longer necessary due to recent advances. New methods of error modeling allow for sequence variants to be distinguished by a single base, generating amplicon sequence variants (ASV) which are comparable to OTU's, but where OTUs are clustered by percentage sequence identity, ASV's correspond to an exact amplicon sequence variant in the sample (56). A major consideration when choosing a 16S rRNA gene sequence analysis pipeline is the degree of damage to the DNA. As mentioned earlier, it is possible to measure this based on global imbalance value (45). The presence of damaged DNA could make methods reliant on ASV generation unsuitable, as an example, SNPs arising as artifacts of the FFPE process would erroneously be recorded as different strains. In these circumstances, the clustering-based OTUs may prove the more reliable method. Several of these are contained within the QIIME environment, such as Usearch (57). Samples can be analyzed with both clustering and ASV methods, and a comparison of the number of observed species identified could inform the user on the level of damage. When combined with experimental knowledge for example laboratory based culturing from tumors, a large amount of closely related species reported by ASV generating methods but not clustering methods could indicate unrepaired DNA damage.

## Best Practice

As there is currently no established best practice for sequence analysis of bacteria residing in tumor tissue, fresh or formalin fixed, the primary objective of this article is the proposal of such. The section below, along with Figure 3, summarizes a methodology that falls in line with what is currently accepted for 16S rRNA gene sequence analysis, incorporating sample-specific modifications as outlined earlier.

### Pre-analysis

**Figure 4:**
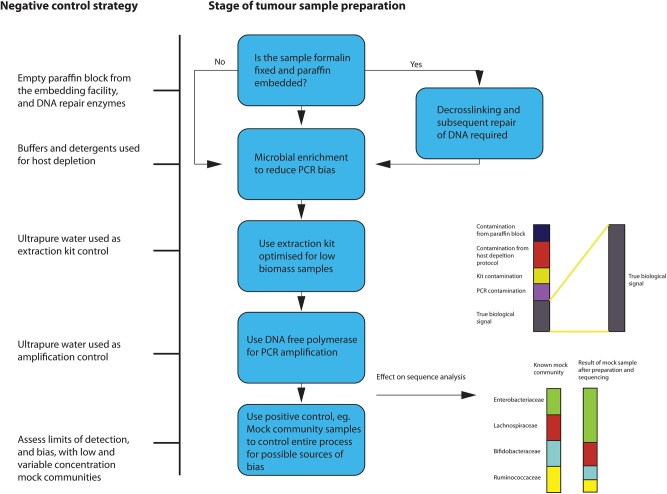
Overview of suggested sample preparation with appropriate control for contamination and bias.

During the extraction process, microbial enrichment, and DNA repair, if the sample originates from FFPE tissue, should be carried out if possible. Since, in low biomass samples, the biological signal can be significantly altered by the presence of contaminants, extreme “aseptic” care must be taken when preparing the samples for sequencing. A variety of controls to account for introduction of contamination should be used. Given the documented effects that a lack of controlling for contamination has had on previous tumor microbiota studies, this is of paramount importance. Eisenhofer et al. recently published a comprehensive description of a robust strategy to control for contamination in low biomass studies (58). This suggests using a variety of negative controls to assess the degree of contamination introduced during the processes of sampling, DNA extraction and amplification. Positive controls are also recommended, such as mock communities of known microbial composition and amplification controls. This should be adhered to when sequencing from FFPE tissues, with some additional steps as outlined below in Figure 4.

### Bioinformatic Analysis

**Figure 5:**
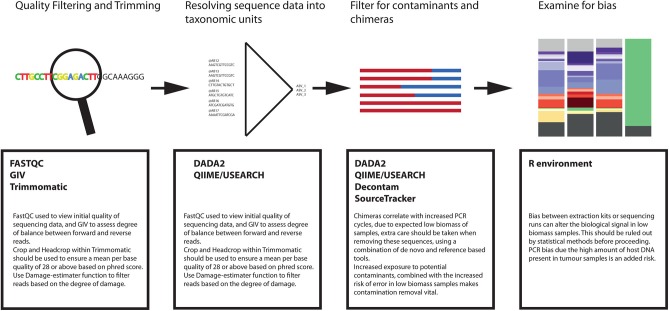
Suggested bioinformatics workflow for bacterial sequence analysis from tumor tissue.

Figure 5 summarizes the key points outlined previously in this article in relation to the required modifications to a bioinformatic pipeline required to ensure high quality reproducible analysis.

## Possible Improvements

Typically, hypervariable regions within the 16S rRNA gene fragment are targeted by primers, the most commonly targeted is the V3–V4 region as it is thought to provide the best resolution. While this method is effective to genus level in most cases, species level classification is often unsuccessful. An obvious solution would be to simply increase the length of the reads, as sequencing technologies such as Oxford Nanopore sequencing are capable of producing reads that are hundreds of kb in length, it should be straightforward to simply sequence the entire 16S rRNA gene fragment (59). Specifically in the case of sequencing samples from formalin fixed samples however, this is currently not possible, as the DNA will often be fragmented, preventing long read sequencing. A potential solution to this is to combine multiple, independently sequenced short regions within the 16S rRNA gene fragment. One way this has been implemented is in the Short Multiple Regions Framework (SMURF) method, by Fuks et al. (60). This entails independent amplification and sequencing of multiple regions along the gene fragment, these are then computationally combined to provide a significantly more accurate assessment of the microbial community. When tested on a Human Microbiome Project “Mock” community, it was found that the increase in resolution was a function of the number of regions analyzed. Using two different regions resulted in a two-fold increase in resolution, while using 6 resulted in a ~100 fold increase in resolution (60).

A further improvement not directly related to the bioinformatic analysis but to sample preparation. As was mentioned earlier, while there are extraction kits for DNA in FFPE tissues, these do not take into account damage that may have occurred to the DNA during the fixation process, or the high ratio of host to bacterial DNA. To make metagenomic analysis of tumor samples from FFPE tissues a reliable and crucially reproducible option, there is a genuine need for the establishment of a validated protocol to extract bacterial DNA from FFPE tissues, repair the damage, and deplete the host DNA.

## Concluding Remarks

In conclusion, taking advantage of the presence of bacteria in tumors has the potential to contribute to cancer treatments in the future. As the field is still in its infancy, it is important for data to be as truly representative as possible. It is the objective of this article to provide a guideline for more effective bioinformatic analysis of the tumor microbiota in future.

## Author Contributions

SW, MT, and MC devised and prepared the manuscript. All authors read and approved the final manuscript.

### Conflict of Interest

The authors declare that the research was conducted in the absence of any commercial or financial relationships that could be construed as a potential conflict of interest.
